# The first salen-type ligands derived from 3',5'-diamino-3',5'-dideoxythymidine and -dideoxyxylothymidine and their corresponding copper(II) complexes

**DOI:** 10.1186/1860-5397-2-17

**Published:** 2006-08-25

**Authors:** Daniel Koth, Michael Gottschaldt, Helmar Görls, Karolin Pohle

**Affiliations:** 1 Institute for Organic and Macromolecular Chemistry, FSU Jena, Humboldtstrasse 10, 07743 Jena, Germany; 2 Institute for Inorganic and Analytical Chemistry, FSU Jena, Lessingstrasse 8, 07743 Jena, Germany; 3 Biocatalysis and Organic Chemistry, Delft University of Technology, Julianalaan 136, 2628 BL Delft, The Netherlands

## Abstract

**Background:**

There are many nucleoside metal complexes known. According to observations made, only very few of them reveal their central ion to be co-ordinated by the sugar part of their molecules. The regio- and stereospecific exchange of the hydroxyl groups at the sugar moiety by chelating units improves its complexation ability and should give access to a new class of chiral ligands.

**Results:**

In this paper we present the synthesis of 3',5'-diamino substituted thymidines with *ribo*- as well as *xylo*-configuration and the preparation of copper(II) complexes derived from their corresponding Schiff bases. Starting from thymidine, the amino derivatives were prepared in a three and four step reaction sequence respectively. The absolute configuration of the ligands was proved by the three-bond ^1^H-^1^H spin spin coupling constants ^3^J obtained by NMR-studies. Condensation of the amino derivatives with salicylic aldehydes resulted in the corresponding diimines, which represent a new class of chiral salen-type ligands. All ligands formed uncharged stable copper(II) complexes. The structure of 3',5'-bis(3,5-di-*tert*-butylsalicylaldiminato)-3',5'-dideoxyxylothymidine-copper(II) could be determined by single crystal X-ray structure analysis. The copper centre in this complex has distorted tetrahedral coordination geometry.

**Conclusion:**

For the synthesis of 3',5'-diamino-3',5'-dideoxy thymidines with *xylo*- as well as *ribo*-configuration an effective synthesis pathway has been developed. Their corresponding salicylidene imines form stable coordination compounds with copper(II) ions. They represent the first salen type complexes of nucleosides with this substitution pattern.

## Background

Complexes of diimino functionalized ligands are often used as catalysts for a wide variety of reactions. Enantioselective synthesis has gained in importance in the last few years, and the development of chiral ligands has become an important field in organic chemistry. Nucleosides provide a stable scaffold containing three (DNA nucleosides) and four (RNA nucleosides) chiral centers respectively. Though the coordination of metal ions to nucleosides is well known, mostly the nucleobase interacts with the metal centre, and the chiral part of the nucleoside is not involved in the coordination. [1–2] Although a lot of sugar based complexes have been described already [3–4], so far, the use of the carbohydrate moiety of nucleosides as a binding group in metal complexes has only been known for very few examples. [5–7] Previously, we reported the synthesis of a 2',3'-diimino functionalized uridine and its use as ligand in a copper(II) complex. [6] As far as we are aware, a metal complex with a 3',5'-diimino substituted nucleoside as a ligand has not been published by another source yet, and neither has its carbohydrate analogue. This study reports the synthesis of 3',5'-diamino-3',5'-dideoxy- and 3',5'-diamino-3',5'-dideoxyxylothymidine, and the copper(II) complexes of their corresponding salicylaldiminato-derivatives.

## Results and Discussion

**Scheme 1:**
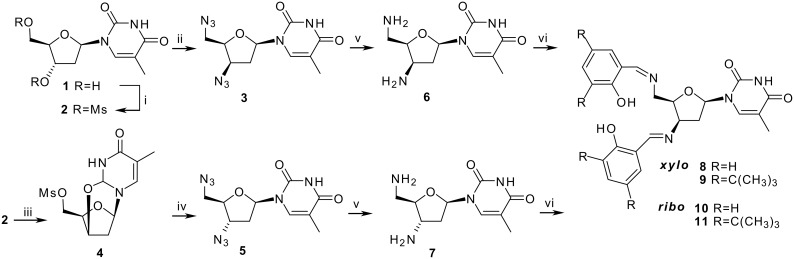
*Reagents and conditions:* i) MsCl, pyridine, 0°C, 15 h, 86%; ii) NaN_3_, DMF, 80°C, 7 d, 80%; iii) Et_3_N, EtOH, reflux, >95%; iv) LiN_3_, DMF, 130°C, 24 h, 97%; v) Pd/C, Hydrogen 5 Atm, EtOH, 95%; vi) salicylic aldehyde, EtOH, reflux.

The most demanding step in the synthesis of the 3',5'-diimino functionalized nucleosides **8**–**11** was the preparation of the diazido derivatives **3** and **5**. The *xylo*-isomer **3** itself has only been described once as a by-product, whose analytical characterization we found had been given insufficiently. [9] Starting with commercially supplied thymidine, and using methanesulfonyl chloride in pyridine, compound **2** was obtained in a yield of 86%. Nucleophilic substitution with activated sodium azide [10] led to the diazide **3** with inverted configuration at C-3' (Scheme 1).

In contrast to this, the synthesis of the diazide **5** with *ribo* configuration seems to be well investigated. [11–12] According to known descriptions the anhydro-derivative **4** had been prepared by converting the mesylate **2** with triethylamine in ethanol. The less active leaving group at C-3' in compound **4** caused low rates of nucleophilic substitution at the desired position. Additional modifications of the reaction condition allowed us to enhance the synthesis rate of **5**. Improved results for step iv could be achieved through to the use of lithium azide instead of the less reactive sodium azide as well as increasing the reaction temperature, which shortened the reaction time. The *ribo* diazide **5** could be obtained in an overall yield of 80%.

For both, the *xylo* diazide **3** as well as the *ribo* diazide **5** an explicit proof of the absolute configuration and a definitive assignment of the NMR-signals has not been published. However, in our case the determination of the absolute configuration is indispensable and important especially for the subsequent ligands **8**–**11**.

To prove the absolute configuration at C-3' in our compounds, a detailed analysis of the three-bond ^1^H-^1^H spin spin coupling constants ^3^J obtained by NMR-studies had been made. Following this, the Karplus relationship was used to determine the dihedral torsion angles. [13–14] When summed up, these angles provide the evidence of structure and configuration. (for details see also: Supporting Information File 1) The obtained results match the expected owing to the synthesis pathway.

After the palladium catalyzed reduction of the azido derivative **5** had gone on for 24 h the resulting amine **7** could be obtained as a pure white solid without need for further purification. However, it appeared more complicated to reduce the azide **3** since the azido-substituent is in a sterically hindered position. Replacing the catalyst by PdO_2_, which forms *in situ* the active Pd species, and raising the reaction temperature as well as lengthening the reaction time led to the desired 3',5'-diamino-3',5'-dideoxy-β-D-*xylo*-thymidine **6** in 90% yield. Although this compound is easy to synthesize, it has not been described yet. Subsequent condensation of **6** and **7** with salicylic aldehyde provides compounds **8**–**11** as crystalline solids.

**Scheme 2:**
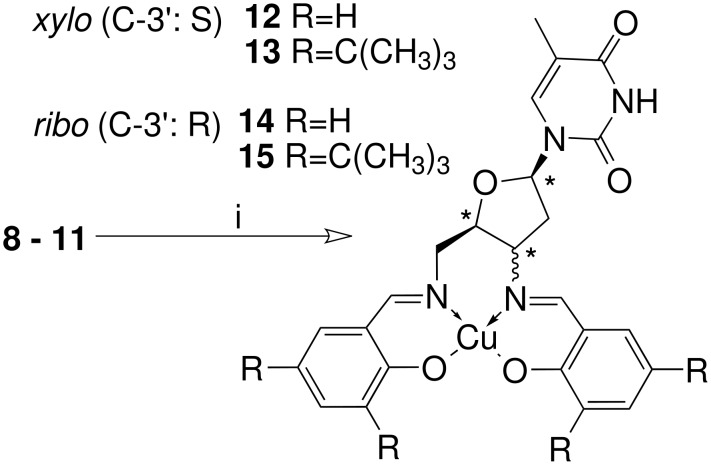
*Reagents and conditions:* i) Cu(OAc)_2_, THF, 5 h RT, >90%.

The Schiff bases **8**–**11** of the 3',5'-diaminothymidines in *ribo*- and *xylo*-configuration represent the first nucleosides bearing salen-type chelating units at these positions. With their synthesis a new class of tetradentate chiral ligands with interesting features could be obtained. The chiral sugar moiety is close to the metal binding site and the *N*-glycosidic bound thymine provides an additional chiral information and steric shielding. After the ligands had been added to a mixture of copper(II) acetate in THF the complexes were formed within a few minutes resulting in a dark green solution (Scheme 2).

**Figure 1:**
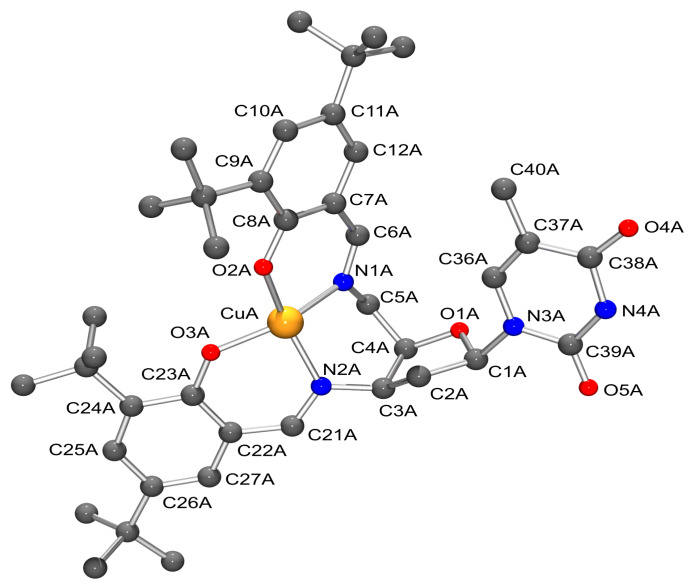
X-ray crystal structure of one of the molecules in **13** with used crystallographic numbering. H-atoms, the solvent molecule and the labels at the *tert*-butylgroups are omitted for clarity. Atomic displacement parameters are drawn at the 50% probability level.

All four ligands **8**–**11** formed uncharged stable copper(II) complexes with double deprotonated ligands as MS experiments had shown. Recrystallization of complex **13** using DMF resulted in crystals suitable for single crystal X-ray structure analysis [15] (For details see also: Figure 1 and Supporting Information File 2).

**Figure 2:**
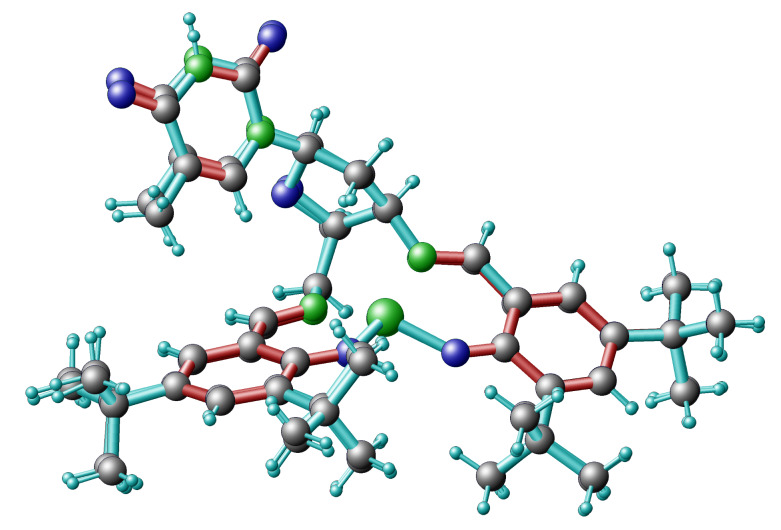
This figure shows an overlay of the two complex molecules contained in the asymmetric unit.

The asymmetric unit of the crystals contained two symmetrically independent molecules of **13** and one solvent molecule, which was not bound to the complex. As seen in the overlay of the molecular structures (see Figure 2), there is only a very small difference in the periphery between the two complex molecules within the asymmetric unit. The thymine unit was not involved in the coordination, neither inter- nor intramolecularly. The coordination sphere of the copper centre had been determined to be distorted tetrahedral (Table 1). The obtained data confirmed the *xylo* configuration of the thymidine in compounds **3** and **6** and supported the NMR experiments.

**Table 1:** Selected bond lengths [Å] and angles [°] of complex 13.

CuA-O2A	1.892(3)	O2A-CuA-O3A	90.33(11)
CuA-O3A	1.885(3)	O2A-CuA-N1A	92.96(13)
Cu-N1A	1.935(3)	O3A-CuA-N2A	93.63(12)
Cu-N2A	1.945(30)	N1A-CuA-N2A	92.13(13)

The dihedral angle at the copper centre between the planes defined by CuA-N1A-O2A and CuA-N2A-O3A is 38.8°. Therefore the copper has a distorted tetrahedral coordination geometry.

All four complexes show the expected ligand to metal charge transfer transitions [16–17] in the UV/VIS spectra around 400 nm, compounds with salicylaldiminato-substituted ligands **12** (366 nm), and **14** (364 nm) shortly below to those of **13** (390 nm) as well as **15** (384 nm). The complexes of the diastereomeric ligands could be obtained in a straightforward synthesis. They possess interesting features especially with regard to chiral catalysts or DNA strand formation. Although located off the metal ion the thymine base may act as a substrate binding site in catalytic reactions, demonstrating that modified nucleosides could act as chiral ligands for transition metal ions. This approach opens up a new class of metal complexes containing ligands based on nucleoside derivatives. The wide variety in this type of ligands in terms of their structural diversity such as the replacement of the thymine base by different functional groups or the immobilization of the complexes by the nucleobase enables the resulting complexes to become promising candidates as catalysts for enantioselective reactions.

## Conclusion

In summary, an effective route for the synthesis of 3',5'-diamino-3',5'-dideoxy thymidines in *xylo*- as well as *ribo*-configuration had been developed. It is notable that the synthesis of both isomers delivered the diastereomeric compounds within three (compound **6**) and four (compound **7**) reaction steps respectively in good yield. The corresponding salicylidene imines formed stable coordination compounds with copper(II) ions, which represent the first salen type complexes of nucleosides with this substitution pattern. These complexes tap the full potential of nucleoside derivatives as chiral ligands for transition metal ions.

## Supporting Information

File 1 Supplementary experimental data: This file contains all experimental methods and analytical data belonging to the compounds described in the article

File 2 Proof of the configuration. Within this file is a short description about how to conclude from the three-bond-coupling constants the absolute configuration of the molecules.
