# Supplementary material for: The first salen-type ligands derived from 3',5'-diamino-3',5'-dideoxythymidine and -dideoxyxylothymidine and their corresponding copper(II) complexes
Source: Beilstein J Org Chem. 2006 Aug 25;2:17. doi: 10.1186/1860-5397-2-17 (PMC1592495; doi:10.1186/1860-5397-2-17)
Supplement: File 1 — Supplementary experimental data: This file contains all experimental methods and analytical data belonging to the compounds described in the article [file Beilstein_J_Org_Chem-02-17-s001.pdf]

Supplementary experimental data:

The first salen-type ligands derived from 3′,5′-diamino-3′,5′-dideoxythymidine and -dideoxyxylothymidine and their corresponding copper(II) complexes

General methods:

Electronic spectra were recorded with a Varian Cary 1 or Cary 5E spectrophotometer at room temperature. IR spectra were measured on a Perkin-Elmer 2000 spectrometer; NMR spectra on a Bruker AC-200, mass spectra were carried out on a Finnigan MAT SSQ 710 or a Finnigan MAT 95XL TRAP, elemental analyses on a Leco CHNS 932.

*Synthesis of 3′,5′-di-O-(methansulfonyl)thymidine **2**.* [1]

To a solution of commercially available thymidine **1** (23.94g, 98.74 mmol) in dry pyridine (250 mL) methanesulfonyl chloride (24 g, 209.5 mmol) was added drop wise at 0°C and stirred for 16 hours. After the reaction has been completed the solution was poured into 600 mL ice water. The formed precipitate was filtered of, washed twice with water and dried in vacuum over phosphorous pentoxide to give the desired product as a white powder in 86% yield (33.8 g). (Found: C, 36.22; H, 4.54; N, 6.98; S, 15.98. $C_{12}H_{18}N_2O_9S_2$ requires C, 36.18; H, 4.55; N, 7.03; S, 16.10%). δ_H (250 MHz; DMSO-d_6) 11.39 (1 H, s, NH), 7.49 (1 H, s, H-6), 6.21 (1 H, t, *J* 7.09, H-1′), 5.29 (1 H, dd, *J* 4.21 and 7.13, H-3'), 4.44 (2 H, d, J 3.82, 2 × H-5′), 4.37 (1H, dd, J 3.78 and 3.09, H-4′), 3.34 (2H, s, 2 × H-2′), 3.32 and 3.25 (6H, s, 2 × -SO_2CH_3), 1.79 (3H, s, -CH_3); δ_C (DMSO-d_6) 163.5 (C-4), 150.3 (C-2), 135.8 (C-6), 110.0 (C-5), 84.0 (C-1′), 80.5 (C-4′), 79.3 (C-3′), 68.4 (C-5′), 37.7 and 36.8 (-SO_2CH_3), 36.0 (C-2′), 11.9 (-CH_3); υ_{max} (ATR, cm^{-1}) 3023, 1712, 1675, 1340, 1321, 1166, 953, 836, 822, 760, 627; MS (DCI water) *m/z* 399 ($[M+1]^+$).

*Synthesis of 3′,5′-Diazido-3′,5′-dideoxyxylothymidine **3**.*

To a solution of 3′,5′-di-*O*-(methanesulfonyl)thymidine **2** 2.79 g (7 mmol) in DMF (25 mL) 4.59 g (70.6 mmol) sodium azide were added and the reaction mixture was heated for 7 days at 65°C. After that, the solvent was removed, the residue was dissolved in chloroform and filtered through a small pad Al_2O_3 to remove the sodium azide, evaporated and the formed viscous oil was purified by column chromatography. The product **3** was obtained in a yield of 80%. (Found: C, 41.42; H, 4.06; N, 39.63. $C_{10}H_{12}N_8O_3$ requires C, 41.10; H, 4.14; N, 38.34%). δ_H (250 MHz; DMSO-d_6) 11.34 (1 H, s, NH), 7.47 (1H, d, *J* 1.2, H-6), 6.07 (1H, dd, *J* 4.0 and 7.8, H-1′), 4.53 (1H, ddd, *J* 2.3, 4.3, 6.6, H-3′), 4.15 (1H, td, *J* 4.7 and 7.3, H-4′), 3.69 (2H, dq, *J* 6.1 and 13.0, 2 × H-5′), 2.75 (1H, ddd, *J* 6.6, 7.7 and 14.6, H-2′), 2.14 (1H, ddd, *J* 2.3, 4.0 and 14.8, H-2′), 1.98 (1H, s, -CH_3); δ_C (DMSO-d_6) 163.6 (C-4), 150.3 (C-2), 135.4 (C-6), 109.5 (C-5), 83.2 (C-1′), 79.7 (C-4′), 60.8 (C-3′), 49.8 (C-5′), 36.8 (C-2′), 12.3 (-CH_3); υ_{max} (ATR, cm^{-1}) 3041, 2093, 1657, 1296, 1082, 864; MS (DEI) *m/z* 292 ($[M]^+$).

*Synthesis of 2,3′-Anhydro-5′-O-(methansulfonyl)thymidine **4**.* [1,2]

5 g of 3′,5′-di-*O*-(methanesulfonyl)thymidine **1** (12.55 mmol) were suspended in 125 mL fresh distilled ethanol. After 12 mL triethylamine were added the reaction mixture was heated under reflux for 30 hours. After two hours of reflux the consistency of the unsolved solid changed from a powder to a more gelatinous form. After the reaction was completed, the solid product was separated by filtration and washed twice with water and diethyl ether to yield 95% of the desired product. (Found: C, 43.63; H, 4.79; N, 9.24; S, 10.53. $C_{10}H_{12}N_8O_3$ requires C, 43.70; H, 4.67; N, 9.27; S, 10.61 %). δ_H (250 MHz; DMSO-d6) 7.57 (1 H, s, H-6), 5.88 (1 H, d, *J* 3.0, H-1′), 5.33 (1 H, s, H-3′), 4.44 (2 H, dd, *J* 4.1 and 7.3, H-4′ and H-5′), 4.19 (1 H, dd, *J* 8.1 and 12.0, H-5′), 3.18 (3 H, s, SO_2CH_3), 2.62 – 2.45 (2 H, m, 2 × H-2′), 1.75 (3 H, s, -CH_3); δ_C (DMSO-d_6) 170.9 (C-4), 153.1 (C-2), 136.6 (C-6), 116.1 (C-5), 86.9 (C-1′), 81.8 (C-4′), 76.9 (C-3′), 68.1 (C-5′), 36.7 (SO_2CH_3), 32.6 (C-2′), 12.9 (-CH_3); υ_{max} (ATR, cm^{-1}) 1658, 1631, 1612, 1527, 1352, 1174, 1136, 970, 802, 780; MS (DEI) *m/z* 302 ($[M]^+$).

*Synthesis of 3′,5′-diazido-3′,5′-dideoxythymidine **5**.* [1]

To a solution of 4.6 g of the anhydro derivative **4** in 60 mL DMF, 5 g lithium azide were added and the reaction mixture was heated at 130°C for 6 hours. After it's cooled down the solvent was evaporated, the residue extracted twice with ethyl acetate from water. The organic layer was dried with sodium sulphate, filtered of and the solvent was evaporated to yield 4.3 g (97%) of the crowd product, which has been purified by flash column chromatography. (Found: C, 41.29; H, 4.15; N, 38.20. $C_{10}H_{12}N_8O_3$ requires C, 41.10; H, 4.14; N, 38.34%). δ_H (250 MHz; $CDCl_3$) 9.91 (1 H, s, NH), 7.27 (1 H, q, *J* 1.2, H-6), 6.13 (1 H, dd, *J* 6.5, H-1′), 4.25 (1 H, td, *J* 5.9 and 6.4, H-3′), 3.93 (1 H, td, *J* 3.6 and 5.6, H-4′) 3.75 (1 H, dd, *J* 3.3 and 13.4, H-5′), 3.56 (1 H, dd, *J* 3.8 and 13.4, H-5′), 2.42 – 1.90 (2 H, m, 2 × H2′) 1.91 (1 H, d, *J* 1.2, CH_3); δ_C ($CDCl_3$) 164.0 (C-4), 150.5 (C-2), 135.5 (C-6), 111.6 (C-5), 85.2 (C-1′), 82.1 (C-4′), 60.4 (C-3′), 51.8 (C-5′), 37.2 (C-2′), 12.6 (-CH_3); υ_{max} (ATR, cm^{-1}) 3041, 2092, 1652, 1264, 1083, 959, 769; MS (EI) *m/z* 292 ($[M]^+$).

*Synthesis of 3′,5′-diamino-3′,5′-dideoxyxylothymidine **6**.*

To a solution of 6.43 g (22 mmol) of 3′,5′-diazido-3′,5′-dideoxyxylothymidine **3** dissolved in 40 mL dried ethanol and 20 mL dried methanol 125 mg PdO_2 were added and stirred for 7 days under 5 atmosphere hydrogen at 40°C. The formed palladium was filtered off and the desired product precipitates after slow evaporation as a white solid. Yield 4.9 g (95%). (Found: C, 49.44; H, 6.84; N, 21.91. $C_{10}H_{16}N_4O_3$ requires C, 49.99; H, 6.71; N, 23.32%). δ_H (250 MHz; D_2O) 7.51 (1H, d, *J* 1.2, H-6), 5.88 (1H, dd, *J* 6.4 and 7.2, H-1′), 3.95 (1H, dt, *J* 6.4 and 5.1, H-4′), 3.64 (1H, dt, *J* 5.1 and 7.3, H-3′), 2.94 (2H, d, *J* 6.6, H-5′/5″), 2.62 (1H, ddd, *J* 7.3, 7.2 and 14.4, H-2′), 1.73-1.87 (4H, m, -CH_3 and H-2″). δ_C (D_2O) 169.3 (C-4), 153.8 (C-2), 137.4 (C-6), 111.3 (C-5), 84.8 (C-1′), 81.4 (C-4′), 50.6 (C-3′), 40.3 (C-5′), 38.6 (C-2′), 11.8 (-CH_3); MS (EI) *m/z* 241 $[M+1]^+$.

Synthesis of 3′,5′-Diamino-3′,5′-dideoxythymidine ***7****.* [1,2]

To a solution of 1 g (3.42 mmol) of 3′,5′-diazido-3′,5′-dideoxyxylothymidine **3** in 40 mL dried ethanol 125 mg Pd/C was added and under 5 atmosphere hydrogen stirred for 48 hours. The palladium was filtered off and the desired product precipitates after slow evaporation as a white solid. Yield 800 mg (97%). (Found: C, 48.24; H, 6.75; N, 22.14. $C_{10}H_{16}N_4O_3$ requires C, 49.99; H, 6.71; N, 23.32%). δ_H (400 MHz; MeOD) 7.54 (1H, q, *J* 1.2, H-6), 6.21 (1H, dd, *J* 5.1 and 7.4, H-1′), 3.67 (1H, dt, *J* 3.7 and 7.0, H-4′), 3.43 (td, 1H, *J* 7.22 and 7.95, H-3′), 3.05 (1H, dd, *J* 3.7 and 13.5, H-5′), 2.92 (1H, dd, *J* 7.0 and 13.5, H-5″), 2.38 (1H, ddd, J 5.1, 8.2 and 13.4, H-2′), 2.22 (1H, td, J 7.3 and 13.8, H-2″), 1.95 (3H, d, *J* 1.1, -CH_3); δ_C (MeOD) 164.3 (C-4), 150.9 (C-2), 136.8 (C-6), 109.8 (C-5), 88.7 (C-1′), 83.5 (C-4′), 52.3 (C-3′), 43.7 (C-5′), 40.8 (C-2′), 12.9 (-CH_3); MS (DEI) *m/z* 241 $[M+1]^+$.

Synthesis of 3′,5′-bis(salicylaldiminato)-3′,5′-dideoxyxylothymidine ***8****.*

To a solution of 299 mg (1.25 mmol) of **6** in 20 mL dry ethanol 305 mg (2.496 mmol) salicylic aldehyde were added and the mixture was refluxed for 2 hours. After the solution has been cooled down to room temperature the formed crystals were filtered of and dried. Yield 0.264 g (47%) yellow-orange needles. (Found: C, 64.23; H, 5.49; N, 12.44. $C_{24}H_{24}N_4O_5$ requires C, 64.28; H, 5.39; N, 12.49%). δ_H (400 MHz; $CDCl_3$) 12.42 (1H, s, -NH), 8.37 and 8.33 (2H, 2 s, =CH-), 6.84 – 7.48 (9H, m, -CH_{aryl} and –CH_{tym}), 6.31 (1H, dd, *J* 4.3 and 7.3, H-1′), 4.45 (1H, dt, J 6.3 and 4.6, H-4′), 4.15 (1H, m, H-3′), 3.97 (1H, dd, J 6.0 and 12.8, H-5′), 3.90 (1H, dd, J 7.0 and 12.7, H-5″), 2.94 (1H, td, J 7.3 and 14.6, H-2′), 2.15 (1H, ddd, J 2.2, 4.2 and 14.6, H-2″), 1.96 (3H, s, -CH_3). δ_C ($CDCl_3$) 167.7 (-CH=N-), 166.7 (-CH=N-), 163.7 (C-4), 160.9 and 160.7 (=C-OH), 150.5 (C-6), 134.9 (C-2), 131.6–133.3 and 117.0–119.3 (C_{aryl}), 111.7 (C-3), 84.3 (C-4´), 81.5 (C-1´), 68.9 (C-3´), 58.9 (C-5´), 40.8 (C-2´), 12.4 (CH_3). MS (ESI) *m/z* 471.1 $[M+Na]^+$. υ_{max} (ATR, cm^{-1}): 3174, 3054, 2963, 2910, 2875, 2829, 1684, 1621, 1577, 1463, 1269, 1046, 1023, 747.

Synthesis of 3′,5′-bis(3,5-di-tert-butyl-salicylaldiminato)-3′,5′-dideoxyxylothymidine ***9****.*

To a solution of 229 mg (0.953 mmol) of **6** in 5 mL ethanol 449 mg (1.916 mmol) 3,5-di-tert-butylsalicylic aldehyde were added and the mixture was refluxed for 2 hours. After cooling down to room temperature the formed crystals were filtered off and dried. Yield 258 mg (42%) of yellow needles. (Found: C, 71.27; H, 8.2; N, 8.22. $C_{40}H_{56}N_4O_5$ requires C, 71.40; H, 8.39; N, 8.33%). δ_H (400 MHz; $CDCl_3$) 12.83 (1H, s, NH), 8.69 (1H, s, OH), 8.47 and 8.40 (2H, 2 s, =CH-), 7.58 (1H, q, J 1.2, H-6), 7.60, 7.41, 7.14, and 7.03 (4H, d, -CH_{aryl}), 6.32 (1H, dd, J 3.7 and 7.8, H-1′), 4.40 (1H, dt, J 4.4 and 6.7, H-4′), 4.15 (1H, m, H-3′), 3.93 (2H, m, H-5′/5″), 2.95 (1H, td, J 7.2 and 14.7, H-2′), 2.18 (1H, ddd, J 1.5, 3.7 and 14.7, H-2″), 2.00 (3H, d, J 1.0, -CH_3), 1.45 and 1.29 (36H, s, $C(CH_3)_3$). δ_C ($CDCl_3$) 168.6 (-CH=N-), 166.9 (-CH=N-), 163.7 (C-4), 157.9 and 160.7 (=C-OH), 150.5 (C-6), 117.4 – 141.0 (C-Ar), 111.5 (C-3), 84.6 (C-4´), 82.3 (C-1´), 69.0 (C-3´), 58.4 (C-5´), 40.9 (C-2´), 35.1 and 34.2 ($C(CH_3)_3$), 31.4 and 29.4 ($C(CH_3)_3$), 12.6 (CH_3). MS

(ESI) *m/z* 695.3 $[M+Na]^+$. υ_{max} (ATR, cm^{-1}): 2954, 2908, 2868, 1708, 1666, 1626, 1472, 1433, 1360, 1270, 1251, 1073.

*Synthesis of 3′,5′-bis(salicylaldiminato)-3′,5′-dideoxythymidine **10**.*
To a solution of 420 mg (1.8 mmol) of **7** in 8 mL ethanol 430 mg (3.5 mmol) salicyl aldehyde were added and the mixture was refluxed for 2 hours. After cooling to room temperature the formed crystals were filtered off and dried. Yield 0.483 g (61%) yellow-orange needles. (Found: C, 63.84; H, 5.66; N, 11.81. $C_{24}H_{24}N_4O_5$ requires C, 64.28; H, 5.39; N, 12.49%). δ_H (250 MHz; $CDCl_3$) 12.53 (1H, s, -NH), 8.48 and 8.44 (2H, 2 s, =CH-), 6.84 – 7.48 (9H, m, $-CH_{aryl}$ and $-CH_{tym}$), 6.36 (1H, dd, J 4.6 and 7.5, H-1′), 4.29 (2H, m, H-3′ and H-4′), 4.02 (1H, m, H-5′), 3.86 (1H, m, H-5″), 2.65 (1H, m, H-2′), 2.39 (1H, m, H-2″), 1.50 (3H, d, J 1.0, CH_3). δ_C ($CDCl_3$) 168.6 (-CH=N-), 166.7 (-CH=N-), 163.6 (C-4), 160.9 and 160.7 (=C-OH), 150.2 (C-6), 135.0 (C-2), 133.1–131.7 and 119.2–117.1 (C_{aryl}), 111.6 (C-3), 84.0 (C-4´), 83.1 (C-1´), 68.0 (C-3´), 58.4 (C-5´), 39.7 (C-2´), 11.7 (CH_3). MS (EI) *m/z* 448.2 $[M]^+$.

*Synthesis of 3′,5′-bis(3,5-di-tert-butyl-salicylaldiminato)-3′,5′-dideoxythymidine **11**.*
To a solution of 340 g (1.4 mmol) of **7** in 8 mL ethanol 643 mg (2.74 mmol) 3,5-di-*tert*-butyl-salicylic aldehyde were added and the mixture was refluxed for 2 hours. After cooling down to room temperature the formed crystals were filtered off and dried. Yield 641 mg (68%) of yellow needles. (Found: C, 70.93; H, 8.53; N, 8.18. $C_{40}H_{56}N_4O_5$ requires C, 71.40; H, 8.39; N, 8.33%). δ_H (250 MHz; $CDCl_3$) 12.82 (1H, s, NH), 9.00 (1H, s, OH), 8.45 and 8.38 (2H, 2 s, =CH-), 7.58 (1H, q, J 1.0, H-6); 7.43, 7.38, 7.13, and 7,01 (4H, d, $-CH_{aryl}$); 6.32 (1H, dd, J 3.6 and 7.8, H-1′), 4.39 (1H, m, H-4′), 4.14 (1H, m, H-3′), 3.92 (2H, m, H-5′/5″), 2.94 (1H, m, H-2′), 2.18 (1H, m, H-2″), 2.00 (3H, d, J 1.0, $-CH_3$), 1.44 and 1.29 (36H, s, $C(CH_3)_3$). δ_C ($CDCl_3$) 168.6 (-CH=N-), 167.8 (-CH=N-), 163.8 (C-4), 157.9 and 157.6 (=C-OH), 150.6 (C-6), 117.4 – 140.9 (C-Ar), 111.6 (C-3), 84.6 (C-4´), 82.3 (C-1´), 69.1 (C-3´), 58.4 (C-5´), 40.9 (C-2´), 35.1, 34.2, 34.1, 31.5, 31.4, 29.4; 29.4 (7 $C(CH_3)_3$), 12.6 (CH_3). MS (EI) *m/z* 672.3 $[M]^+$.

*Synthesis of 3′,5′-bis(salicylaldiminato)-3′,5′-dideoxyxylothymidine-copper(II) **12**.*
100.16 mg of **8** (0.223 mmol) were dissolved in 10 mL THF. Then 40.55 mg (0.223 mmol) copper(II) acetate were added and the resulting dark green solution was stirred at room temperature for 5 hours, filtered of and evaporated under reduced pressure. The residue was dissolved in methanol and precipitated in water, filtered and dried in vacuum yielding 112.3 mg (80%) of **12**. (Found: C, 54.08; H, 4.45; N, 10.11. $C_{24}H_{22}CuN_4O_5{\cdot}H_2O$ requires C, 54.59; H, 4.58; N, 10.61%). υ_{max} (ATR, cm^{-1}) 1691, 1609, 1538, 1447, 1313, 1277, 1060, 733; λ_{max}(MeOH)/nm 225, 241, 274, 366 (logε 4.631, 4.624, 4.517, 3.961); MS (ESI) m/z 532 $[M+Na]^+$, high resolution: found 532.078640000 $C_{24}H_{22}CuN_4O_5Na$ requires 532.078383363.

Synthesis of 3′,5′-Bis(3,5-di-tert-butylsalicylaldiminato)-3′,5′-dideoxyxylothymidine-copper(II) ***13***.

100.11 mg of **9** (0.149 mmol) were dissolved in 10 mL THF. 27.03 mg (0.149 mmol) copper(II) acetate were added and the resulting dark green solution was stirred at room temperature for 5 hours, filtered of and evaporated under reduced pressure. The residue was dissolved in methanol, purified by flash column chromatography using Sephadex LH-20 as stationary phase and methanol as eluent. After evaporation **13** was obtained as a dark green powder in a yield of 100.3 mg (78%). It was recrystallised from DMF and after 5 days at room temperature green crystals were formed suitable for single crystal X-ray structure analysis (Found: C, 64.99; H, 7.5; N, 6.93. $C_{40}H_{54}CuN_4O_5{\cdot}H_2O$ requires C, 65.42; H, 7.41; N, 7.63%). υ_{max} (ATR, cm^{-1}) 2953, 2906, 1692, 1603, 1527, 1464, 1438, 1413, 1360, 1275, 1252, 1169, 1080, 835, 786; λ_{max}(MeOH)/nm 205, 239, 279, 390 (logε 4.591, 4.648, 4.519, 3.970); MS (ESI) m/z 757 $[M+Na]^+$, high resolution: found 756.327660000 $C_{40}H_{54}CuN_4O_5Na$ requires 756.328784324.

X-ray crystal structure of **13**. The intensity data for the compounds were collected on a Nonius KappaCCD diffractometer, using graphite-monochromated Mo-K_α radiation. Data were corrected for Lorentz and polarization effects, but not for absorption effects [3,4]. The structure was solved by direct methods (SHELXS [5]) and refined by full-matrix least-squares techniques against Fo^2 (SHELXL-97 [6]). The hydrogen atoms at N4 of imido-groups of both symmetry independent molecules were located by difference Fourier synthesis and refined isotropically. All other hydrogen atoms were included at calculated positions with fixed thermal parameters. All nonhydrogen atoms were refined anisotropically [6]. XP (SIEMENS Analytical X-ray Instruments, Inc.) was used for structure representations.

Crystal Data for **FO2762** [7]: $C_{40}H_{54}CuN_4O_5{\cdot}C_3H_7NO$, Mr = 807.51 $gmol^{-1}$, green prism, size 0.03 x 0.03 x 0.03 mm^3, triclinic, space group P1, a = 9.7114(3), b = 10.9292(3), c = 20.8652(6) Å, α = 96.497(2), β = 99.426(2), γ = 91.589(2) °, V = 2168.2(1) $Å^3$, T= -90 °C, Z = 2, $\rho_{calcd.}$ = 1.237 gcm^{-3}, μ (Mo-K_α) = 5.54 cm^{-1}, F(000) = 862, 15557 reflections in h(-12/11), k(-13/14), l(-26/27), measured in the range 1.99° $\leq \Theta \leq$ 27.51°, completeness Θ_{max} = 99 %, 15557 independent reflections, 12123 reflections with $F_o > 4\sigma(F_o)$, 1029 parameters, 3 restraints, $R1_{obs}$ = 0.050, wR^2_{obs} = 0.100, $R1_{all}$ = 0.077, wR^2_{all} = 0.114, GOOF = 1.002, Flack-parameter -0.002(9), largest difference peak and hole: 0.420 / -0.395 e $Å^{-3}$.

Synthesis of 3′,5′-bis(salicylaldiminato)-3′,5′-dideoxythymidine-copper(II) ***14***.

100.01 mg of **10** (0.223 mmol) were dissolved in 10 mL THF. Then 40.50 mg (0.223 mmol) copper(II) acetate were added and the resulting dark green solution was stirred at room temperature for 5 hours, filtered of and evaporated under reduced pressure. The residue was dissolved in methanol and precipitated in water, filtered of and dried in vacuum to yield 109.3 mg (78%) of **14**. (Found: C, 54.08; H, 4.45; N, 10.11. $C_{24}H_{22}CuN_4O_5{\cdot}H_2O$ requires C, 54.59; H, 4.58; N, 10.61%). υ_{max} (ATR, cm^{-1}) 1693, 1652, 1610, 1538, 1469, 1445, 1317, 1062, 758; λ_{max}(MeOH)/nm 224, 243, 271, 364 (logε

4.618, 4.573, 4.484, 3.997); MS (ESI) m/z 532 [M+Na]$^+$, high resolution: found 532.079140000 $C_{24}H_{22}CuN_4O_5Na$ requires 532.078383363.

Synthesis of 3′,5′-bis(3,5-di-tert-butylsalicylaldiminato)-3′,5′-dideoxythymidine-copper(II) ***15****.*

100.73 mg of **11** (0.149 mmol) were dissolved in 10 mL THF. Then 27.03 mg (0.149 mmol) copper(II) acetate were added and the resulting dark green solution was stirred at room temperature for 5 hours, filtered of and evaporated under reduced pressure. The residue was dissolved in methanol, purified by flash column chromatography using Sephadex LH-20 as stationary phase and methanol as eluent and finally dried in vacuum yielding 114.3 mg (89%) of **15**. (Found: C, 64.2; H, 7.23; N, 6.54. $C_{40}H_{54}CuN_4O_5 \cdot H_2O$ requires C, 65.42; H, 7.41; N, 7.63%). υ_{max} (ATR, cm^{-1}) 2954, 2901, 1699, 1604, 1530, 1464, 1420, 1391, 1413, 1254, 1252, 1172, 1070, 1042, 834, 787; λ_{max}(MeOH)/nm 230, 277, 311, 384 (logε 4.298, 4.172, 3.595, 3.676); MS (ESI) m/z 735 [M+1]$^+$, 757 [M+Na]$^+$, high resolution: found 756.327760000 $C_{40}H_{54}CuN_4O_5Na$ requires 756.328784324.

1. Lavandera I, Fernandez S, Ferrero M, Gotor V: *J Org Chem* 2001, **66:** 4079-4082.
2. Chiba J, Tanaka K, Ohshiro Y, Miyake R, Hiraoka S, Shiro M, Shionoya M: *J Org Chem* 2003, **68:** 331-338.
3. COLLECT, Data Collection Software; Nonius BV, Netherlands, 1998.
4. Otwinowski Z, Minor W: **Processing of X-Ray Diffraction Data Collected in Oscillation Mode**. In *Methods in Enzymology* 276, *Macromolecular Crystallography Part A*. Edited by Carter CW, Sweet RM., Academic Press; 1997:307-326.
5. Sheldrick GM: *Acta Crystallogr Sect A* 1990, **46:** 467-473.
6. Sheldrick G M SHELXL-97 (Release 97-2), 1997, University of Göttingen, Germany
7. CCDC-603388 (FO2762) contains the supplementary crystallographic data for this paper. These data can be obtained free of charge via http://www.ccdc.cam.ac.uk/conts/retrieving.html
