# Supplementary material for: The first salen-type ligands derived from 3',5'-diamino-3',5'-dideoxythymidine and -dideoxyxylothymidine and their corresponding copper(II) complexes
Source: Beilstein J Org Chem. 2006 Aug 25;2:17. doi: 10.1186/1860-5397-2-17 (PMC1592495; doi:10.1186/1860-5397-2-17)
Supplement: File 2 — Proof of the configuration. Within this file is a short description about how to conclude from the three-bond-coupling constants the absolute configuration of the molecules. [file Beilstein_J_Org_Chem-02-17-s002.pdf]

Prove of the configuration

While spectral data like MS, elemental analysis and NMR-shifts are similar for both isomers, only the three-bond ^1H-^1H spin spin coupling constant ^3J can prove the absolute configuration. The Karplus relationship [1,2] $^3J_{HH} = P_1cos^2\Phi + P_2cos\Phi + P_3$ describes the dependency of the vicinal coupling constant $^3J_{HH}$ on the proton-proton torsion angle Φ. The pentose ring in nucleosides is kept in a conformational equilibrium between N- and S-type conformers [3,4]. Experimental coupling constants represent time-averaged values of the couplings of both conformers and their population in equilibrium.

We want to provide the evidence of the absolute configuration at C-3′ of the epimeric compounds **3** and **5**. In this case, the observed coupling constants can be handled as time averaged values of the dihedral torsion angles, since the individual conformers are negligible.

The Karplus equation is implemented in the MestRe-J program, which can be used to assign a coupling constant to a torsion angle and vice versa [5]. For each coupling constant the Karplus relationship delivers four results for the torsion angle. The difference to 180° for each two angels delivers equal absolute values. As a side condition, the sum or the difference of the dihedral angles from one proton to those protons of the vicinal carbon has to be 120°. This side condition delivers the correct angles. For a given molecule, the knowledge of all dihedral torsion angles provides the evidence of structure and, in this case, the evidence of the absolute configuration.

In Table 1 the decisive calculated angles for compound **3** and **5** are given.

Table 1 Selected ^3J-coupling constants and calculated HCCH-torsion angles of **3** and **5**

	xylo-isomer **3**		*ribo*-isomer **5**	
	J [Hz]	Φ [°]	J [Hz]	Φ [°]
H1′-H2′	7.8	11	7.1	21
H1′-H2″	4.0	120	6.4	137
H3′-H2′	6.6	27	5.5	131
H3′-H2″	2.3	106	7.7	13
H3′-H4′	4.5	45	5.5	131
H4′-H5′	7.3	19	5.7	35
H4′-H5″	4.8	127	4.1	121

The calculation of the torsion angles Φ has been performed by using the Karplus equation

$^3J_{HH} = P_1cos^2\Phi + P_2cos\Phi + P_3$ with the parameter set P_1 7.76, P_2 -1.10, P_3 1.40.

1. Karplus M: *J Chem Phys* 1959, **30:** 11-15.
2. Karplus M: *J Am Chem Soc* 1963, **85:** 2870-2871.
3. Altona C, Sundaralingam M: *J Am Chem Soc* 1972, **94:** 8205-
4. Altona C, Sundaralingam M: *J Am Chem Soc* 1973, **95:** 2333-
5. Navarro-Vázquez A, Cobas JC, Sardina FJ: *J Chem Inf Comput Sci* 2004, **44**, 1680-1685.
